# A Distinct Endocytic Mechanism of Functionalized-Silica Nanoparticles in Breast Cancer Stem Cells

**DOI:** 10.1038/s41598-017-16591-z

**Published:** 2017-11-24

**Authors:** Jiadong Sun, Yajing Liu, Min Ge, Guoqiang Zhou, Wentong Sun, Dandan Liu, Xing-Jie Liang, Jinchao Zhang

**Affiliations:** 1 Key Laboratory of Medicinal Chemistry and Molecular Diagnosis of the Ministry of Education, Hebei University, Baoding, 071002 People’s Republic of China; 2 College of Chemistry and Environmental Science, Chemical Biology Key Laboratory of Hebei Province, Hebei University, Baoding, 071002 People’s Republic of China; 3 CAS Key Laboratory for Biological Effects of Nanomaterials and Nanosafety, National Center for Nanoscience and Technology, Beijing, 100190 People’s Republic of China

## Abstract

Nanoparticles provide new fields for life medical science application, including targeted-drug delivery and cancer treatment. To maximize the delivery efficiency of nanoparticle, one must understand the uptake mechanism of nanoparticle in cells, which may determine their ultimate fate and localization in cells. Recently, the proposed-cancer stem cell (CSC) theory has been attracted great attention and regarded as new targets for the new nanodrug developmet and cancer therapies. The interaction between nanoparticles and cancer cells has been extensively studied, but the uptake mechanism of nanoparticles in CSCs has received little attention. Here, we use the pharmacological inhibitors of major endocytic pathways to study the silica nanoparticle (SiNP) uptake mechanisms in the human breast adenocarcinoma cell line (MCF-7) and MCF-7-derived breast cancer stem cells (BCSCs). The results demonstrate that the uptake of SiNPs, particularly amino-functionalized SiNPs, in MCF-7 cells is strongly affected by the actin depolymerization, whereas BCSCs more strongly inhibit the amino-functionalized SiNP uptake after the scavenger receptor disruption. These findings indicate a distinct endocytic mechanism of functionalized SiNPs in BCSCs, which is significant for designing ideal nanosized drug delivery systems and improving the selectivity for CSC-targeted therapy.

## Introduction

Nanoparticles (NPs) are vital tools in the developing field of biology and nanomedicine; they provide novel ideas for life medical science application, including drug delivery in cancer treatment^1–3^ and gene therapy^4,5^. These NPs enable specific modifications to bind to the targeted cell plasma membranes and enter into cytoplasm or nuclear with longer circulation half-lives and reduced toxicity of the normal tissue. To improve the therapeutic efficacy of nanomedicine, a thorough understanding of NPs uptake mechanisms in cells is required to strengthen the delivery efficiency^6^. Especially, understanding the uptake mechanisms by which NPs are delivered and entered into cell can supply delivery strategies with high targeting efficiency and minimal side effect^7^.

Breast cancer has different subtypes, is regarded as malignant neoplasms with a multidrug-resistant property and high lethality rate worldwide^8^. The multidrug-resistant of a cancer is considered related to small populations of cancer stem cells (CSCs) in the tumors. The proposed-CSC theory indicates that a small population of tumor cells has the ability of self-renewal, cancer-initiating, differentiation and metastasis. CSCs have higher chemotherapeutic resistant ability than most differentiated cancer cells due to the higher expression of drug resistance and anti-apoptotic genes than differentiated cells^9^. If so, a very small number of CSCs can preferentially survive from chemotherapy, even in the case where an apparently suppression of the tumors was observed. This hypothesis is consistent with the studies that chemotherapies that efficiently suppress the tumor reformation rarely inhibit metastasis. In this, CSC-targeted therapy is destined to be a core to development effective anticancer therapeutics. Nanomedicine has an enormous potential in the exploration of CSC-targeted drugs, development of novel gene-specific drugs, controlled drug delivery and release and diagnostic modalities^10,11^. However, the efficiency of nano-based therapy targeted to CSCs is far lower than those targeted to cancer cells^12^. To maximze the efficiency of NP delivery to CSCs, we must understand the uptake mechanisms by which NPs are internalized by CSCs, which possiblely determines their final sub-cellular fate, localization in cells, and efficacy of the cancer treatment. In recent years, scientists have been investigating different mechanisms to understand the cellular internalization processes of NPs with different sizes, shapes, surface charges, and surface chemistry in living cancer cells^13^, which includes clathrin-mediated (CME) and caveolae- and clathrin-independent endocytic mechanism, and phagocytosis. However, the cellular internalization processes of NPs into CSCs are not clear. Understanding the mechanisms of NP cellular internalization may be significant to develop ways to let NPs enter to the nucleus or other organelles for high curative effect or directly deliver nanomedicine to the lesion site by specific surface modification. Recently, inorganic-based nanocarriers (such as silica nanoparticles, SiNPs) have major breakthroughs on the morphology control, temporal control, and surface modification, which provided a great potential for the drug delivery^14^. It has reported that the surface of SiNPs can be easily functionalized with a specific group for targeted release of drugs or genes, which highlight SiNP as potential vehicle for therapeutic applications in biomedical science^15^. In our work, the major endocytic pathways are investigated to understand the carboxyl- and amino-functionalized SiNP uptake mechanisms in MCF-7 and MCF-7-derived CSCs (BCSCs) using seven pharmacological inhibitors. The inhibitors examined in this work are as follows: genistein, which inhibits tyrosine kinases in caveolae-mediated endocytosis^16^; chlorpromazine (CPZ), an inhibitor of the clathrin disassembly and receptor recycling to the plasma membrane during CME^17^; nocodazole, a microtubule-disturbing agent^18^; cytochalasin D, disturbs the actin filaments in cells^18^; Dynasore, which is an inhibitor of dynamin function^7^; Nystain, which interacts with cholesterol^7^; and Poly-I, which is an inhibitor of scavenger receptor^19^. Specially, we determined whether carboxyl- and amino-functionalized SiNPs showed different effects on the cell uptake. The cellular internalization of SiNPs was determined by confocal microscope and transmission electron microscope (TEM) imaging. The inhibition of SiNP uptake rate in the MCF-7 and BCSCs was quantified by flow cytometry relative to the control groups in the absence of pharmacological inhibitors. Importantly, these SiNPs have different uptake mechanisms in MCF-7 and BCSCs, which provide a potential platform for the CSC targeting drug delivery. The result has significance in the design and functionalization of nano-based drug delivery for CSC-targeted therapy.

## Results

### Characterization of Dye-Loaded Functionalized-SiNPs

**Figure 1:**
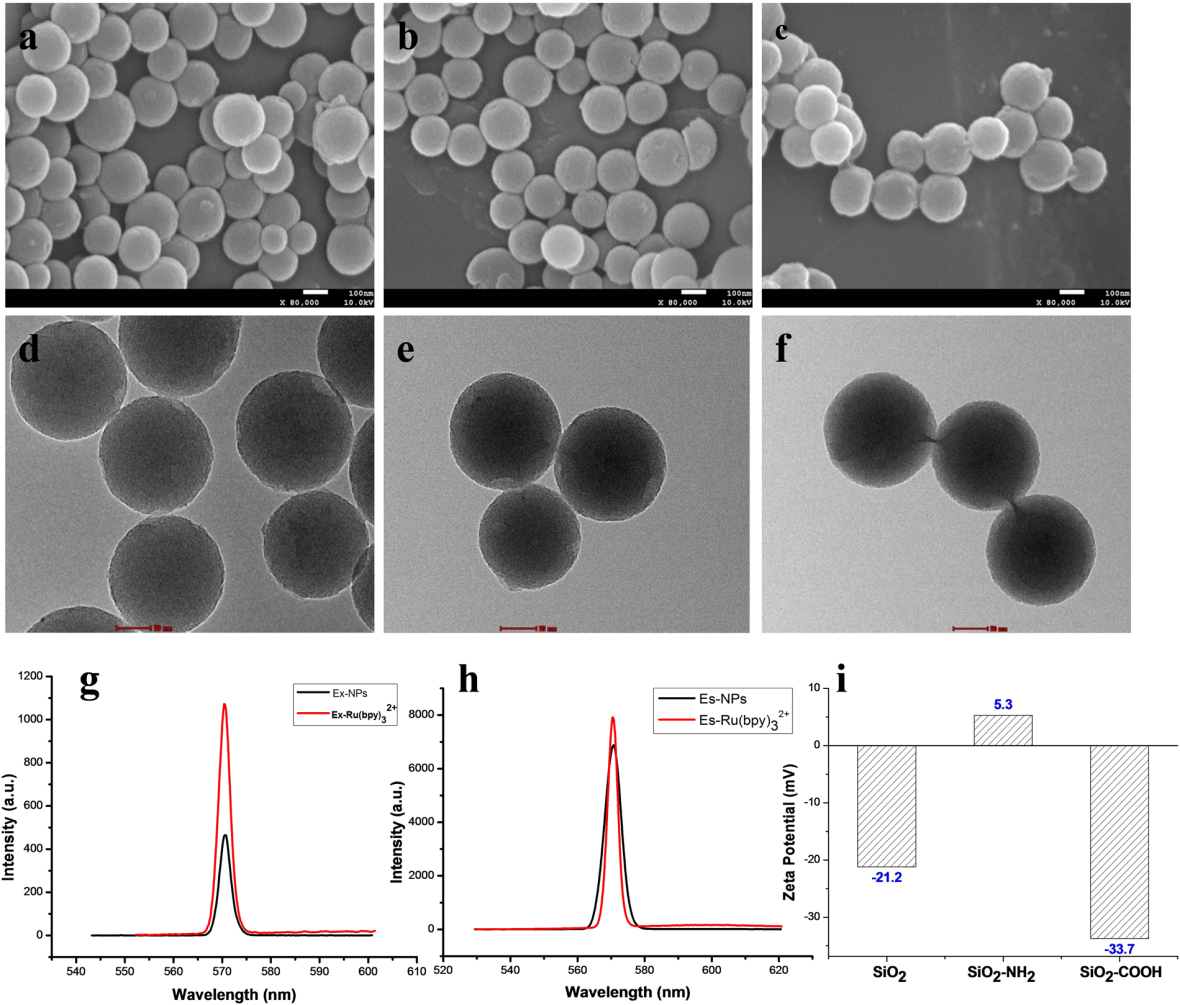
Representative SEM and TEM images of SiNPs with the (**a** and **d**) -OH group, (**b** and **e**) -NH_2_ group, and (**c** and **f**) -COOH group; (**g** and **h**) Fluorescence spectra of SiNPs; (**i**) zeta potential of SiNPs.

SiNPs with different surface groups were successfully prepared, and the average size and shape were determined by TEM and SEM by measuring the size of approximately 100 ± 10 nm (Fig. 1a–f). The amino- and carboxyl-functionalized SiNPs were characterized by IR (Figure S1). Because SiNPs were encapsulated with Ru(bpy)_3_^2+^, their emission wavelengths were verified using a fluorescent spectrometer. SiNPs that were excited at 572 nm had the peak of emission at 570 nm (Fig. 1g and h). Figure 1i shows that the zeta potential of the unfunctionalized SiNPs (SiNPs-OH) was approximately −21.2 mV, i.e., in the absence of an -NH_2_ group and -COOH. However, when functionalized with the -NH_2_ group or -COOH group, the zeta potential was approximately 5.3 mV and −33.7 mV, respectively.

### BCSCs Expressed Markers of Various Stem Cells

**Figure 2:**
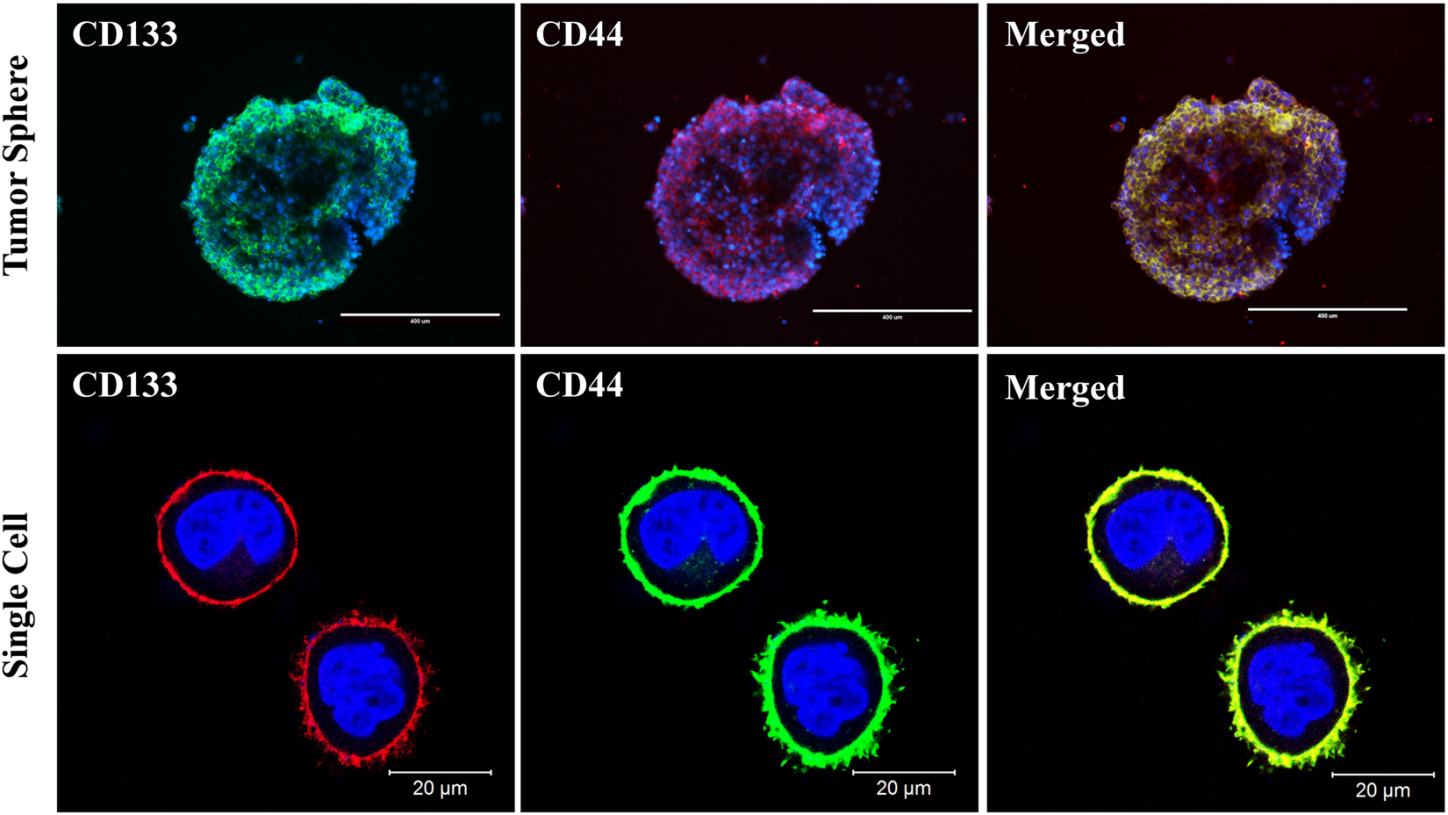
Immunofluorescence detection of the stemness marker expression in the tumor sphere and single BCSC.

The tumor spheres and single BCSC (digested from the tumor sphere), which were cultured and passaged with completed culture medium, could maintain a stable morphological phenotype. Immunofluorescence staining shows that the tumor-sphere-derived BCSCs could stably express cancer stem cell markers CD44 and CD133 (Fig. 2).

### *In vivo* Tumorigenesis of BCSCs

**Figure 3:**
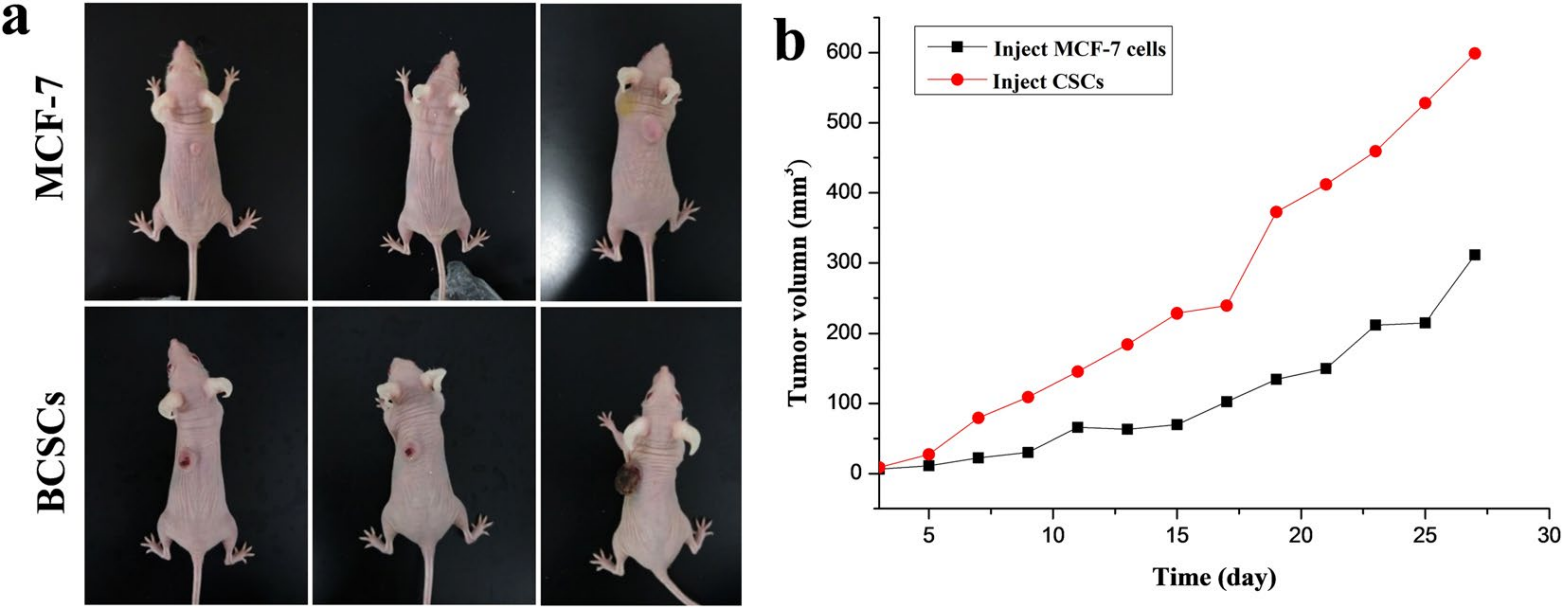
Tumor formation in nude mice after the injection of (**a**) BCSC and (**b**) MCF-7.

As shown in Fig. 3, palpable tumors were visible after a week in the BCSC-injected mice, whereas a bubble growth was observed in MCF-7-injected mice. Additionally, the tumor formation of the enriched BCSCs was faster, which resulted in a sharper tumor growth than that observed after the injection of MCF-7 cells. These data suggest that the BCSC can initiate breast cancer in nude mice.

### Interactions of SiNPs with MCF-7 cells and BCSCs

**Figure 4:**
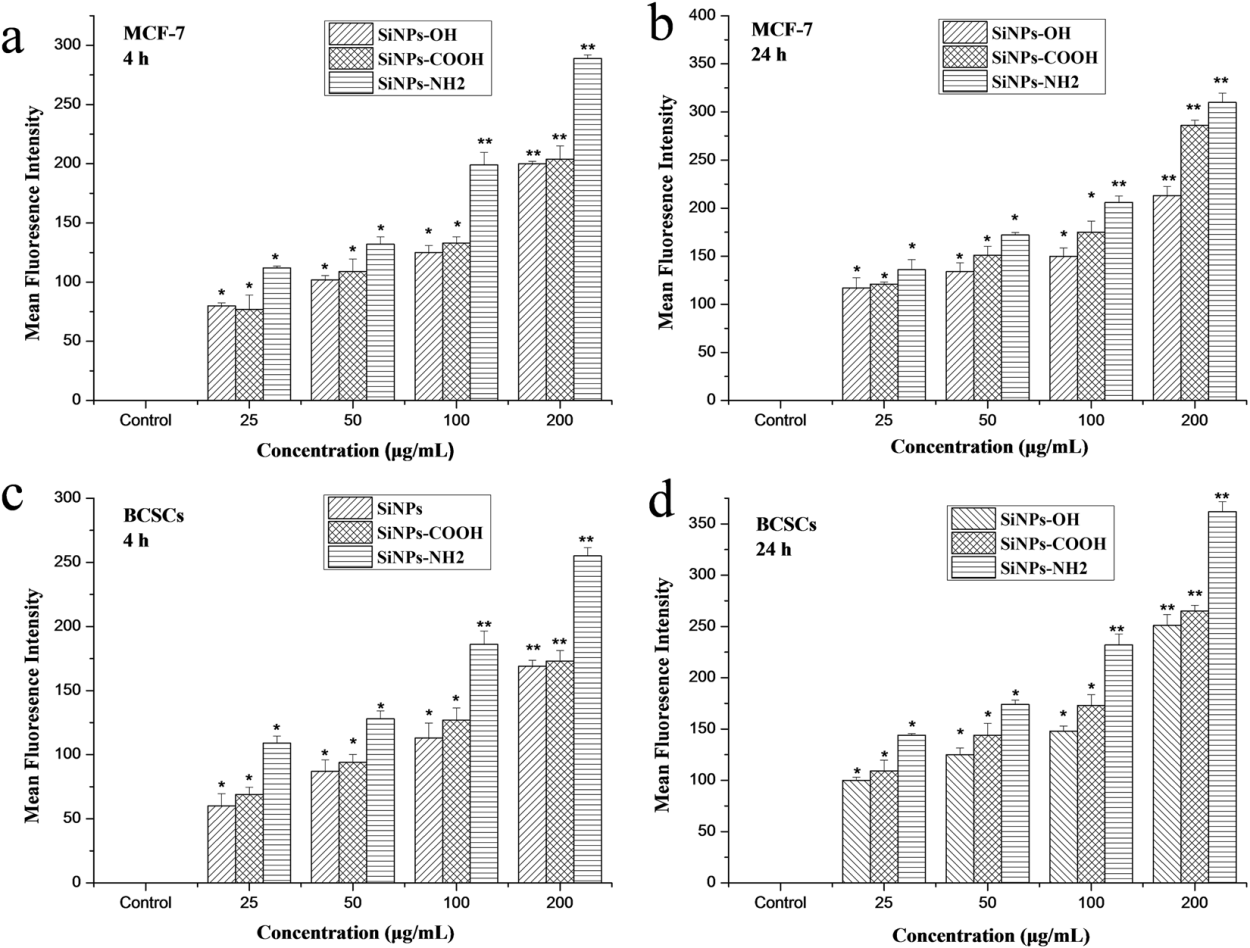
MFI of at least 10,000 (**a** and **b**) MCF-7 cells or (**c** and **d**) BCSCs, which was analyzed by FCM without or with SiNPs-OH, SiNPs-NH_2_, and SiNPs-COOH treatment for 4 and 24 h. The data are expressed as the mean ± SD; n = 5 of one of 3 independent experiments. *p < 0.05; **p < 0.01.

Cells were treated with SiNPs-OH, SiNPs-NH_2_, and SiNPs-COOH at non-cytotoxic concentrations (25, 50, 100, and 200 μg/mL) for 4 and 24 h, respectively (Figure S2). As shown in the flow cytometry, the median fluorescence intensity (MFI) of SiNP-treated cells increased with a time- and dose-dependent manner. Compared to the MFI of non-treated cells, there was a significant difference between control and treatment groups after 4 h and 24 h incubation (Fig. 4). Figure 4 further illustrates that the cellular uptake of SiNPs-NH_2_ in both MCF-7 and BCSCs was significantly higher than those of SiNPs-OH and SiNPs-COOH. In addition, the cell internalization generally increased with the dose.

### SiNPs Localization in Lysosome

**Figure 5:**
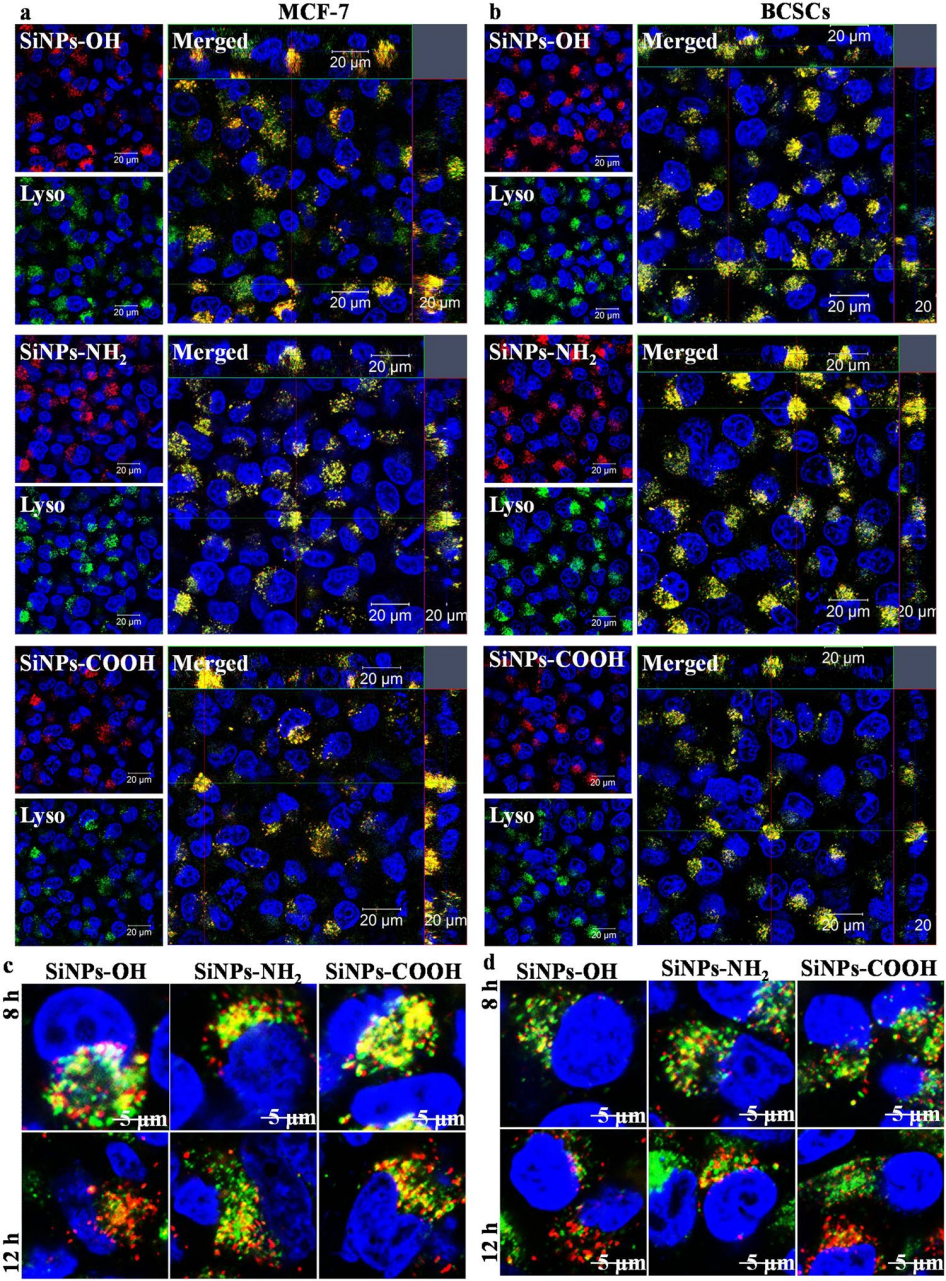
Z-stack images of the SiNPs-OH, SiNPs-NH_2,_ and SiNPs-COOH co-localized with lysosome in (**a**) MCF-7 and (**b**) BCSCs. Lysosome escape of SiNPs in (**c**) MCF-7 and (**d**) BCSCs.

To determine the subcellular localization of SiNPs, the cells were respectively treated with SiNPs-OH, SiNPs-NH_2_, and SiNPs-COOH and subsequently stained with organelle-specific fluorescent markers, which including Hoechst 33342, LysoTracker Green to visualize inside the cells by z-stack imaging. The merged images reveal that SiNPs-OH, SiNPs-NH_2_, and SiNPs-COOH co-localized with lysosomes at 4h (Fig. 5a and b). Figure 5c and d showed that the SiNPs firstly entered the cells and localized in lysosomes, and then the SiNPs escaped into the cytoplasm. The overlap rate of SiNPs with lysotracker reduced to around 70% and 49% after 8 h and 12 h incubation (Figure S6b and S6c), respectively. Magnification images and 3D images of the co-localization of SiNPs-OH, SiNPs-NH_2_, and SiNPs-COOH with lysosome are shown in Figures S3 to S6. The serial z-section images of the cells show the increase in fluorescence intensity from the surface of the cells, which indicates that the NPs were internalized by the cells and not simply bound to their surface.

### Analysis of the Cellular Uptake and Location of SiNPs by TEM

**Figure 6:**
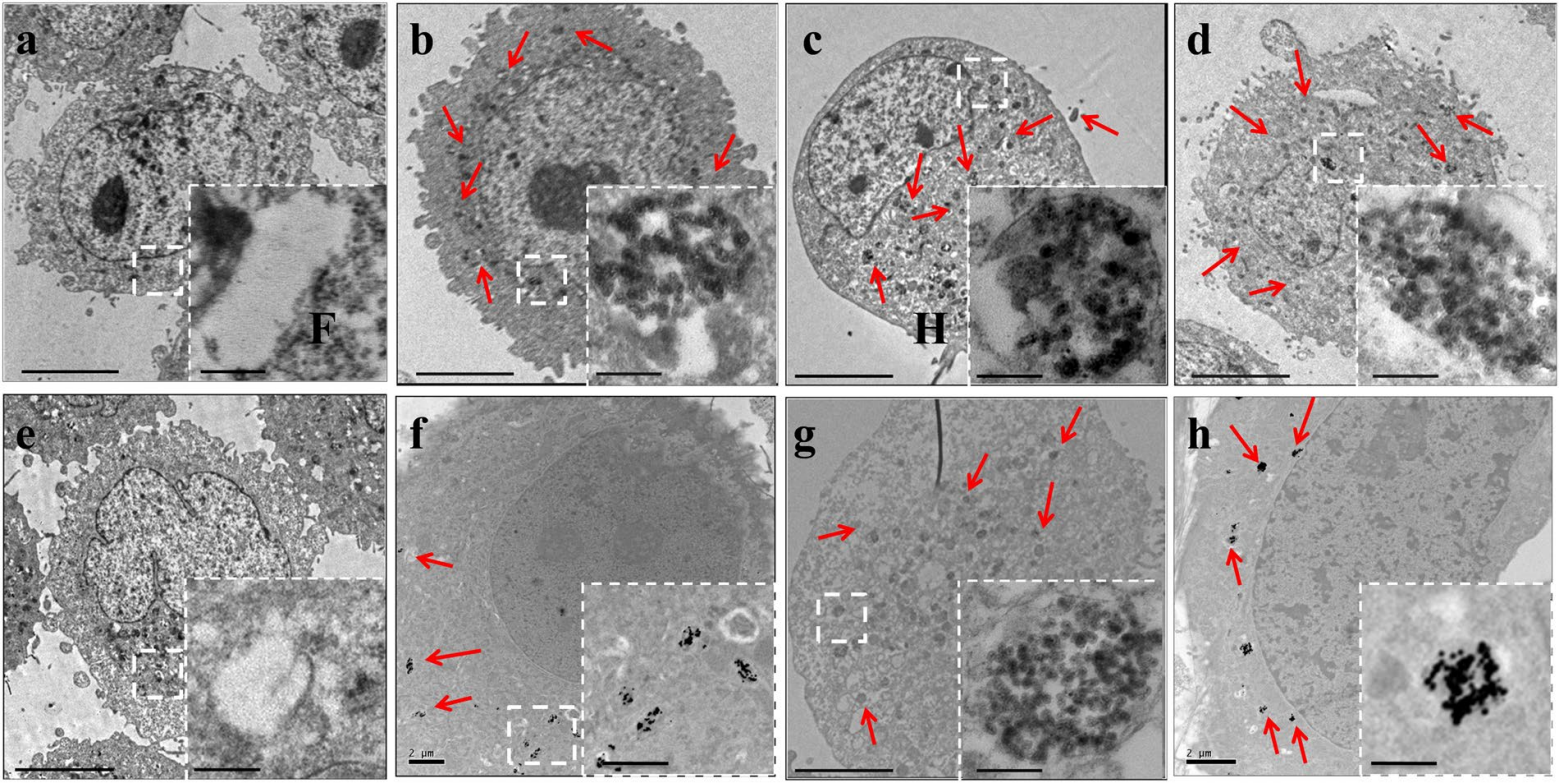
TEM images of (**a–d**) MCF-7 and (**e**–**h**) BCSC cellular uptake of (**b** and **f**) SiNPs-OH, (**c** and **g**) SiNPs-NH_2_ and (**d** and **h**) SiNPs-COOH after 24 h of incubation (scale bar: 5.0 μm). The magnified images of the area are indicated by the white box (scale bar: 500 nm).

To determine whether the SiNPs are taken up by the cells, and if they are, where the SiNPs localize in the cell, TEM images were recorded for MCF-7 and BCSCs that were treated with 200 μg/mL NPs for 24 h. As shown in Fig. 6, most cells were intact, and SiNPs were present in the lysosomes and cytoplasm. Not much cell debris was observed by TEM at this concentration. The nucleus can be identified, and the images consistently indicate that NPs were not present in the nucleus.

### A Distinct Mechanism of SiNP Uptake in BCSCs

**Figure 7:**
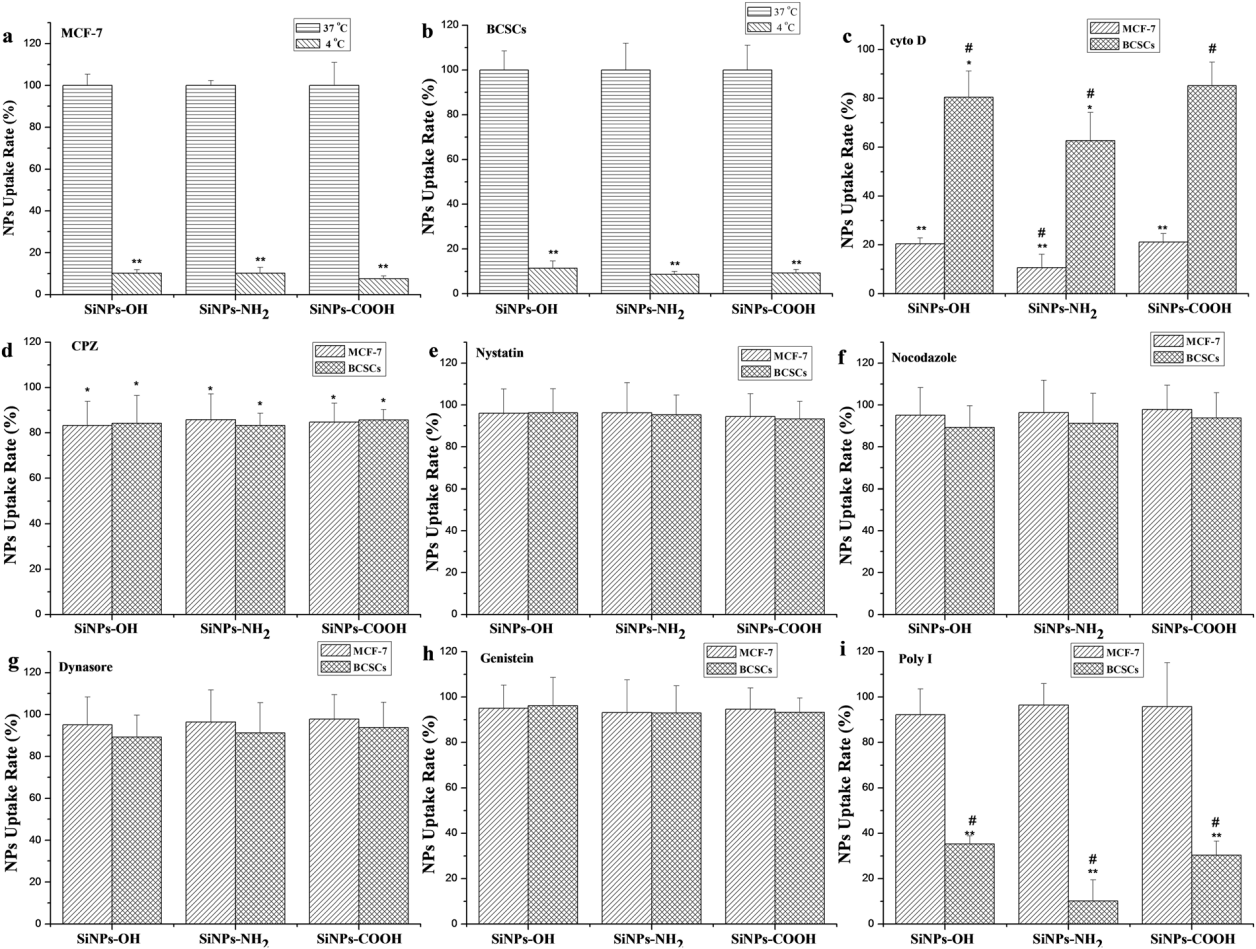
Percentage of cellular uptake of SiNPs-OH, SiNPs-NH_2_, and SiNPs-COOH in MCF-7 and BCSCs in the presence of different endocytic inhibitors. (**a**) and (**b**) Energy-dependent internalization of NPs; (**c**) cyto D; (**d**) CPZ; (**e**) nystatin; (**f**) nocodazole; (**g**) Dynasore; (**h**) Genistein; (**i**) poly I. The values are the mean ± SD from three independent experiments. *p < 0.05; **p < 0.01 BCSCs versus MCF-7 in each group of SiNPs. ^#^p < 0.05 SiNPs-OH versus SiNPs-NH_2_ versus SiNPs-COOH in each cells type.

To understand the major endocytic pathways, the cells were treated with seven inhibitors: CPZ, nystatin, Poly-I, dynasore hydrate, cyto D, nocodazole, and genistein. Prior to the use of inhibitors, the energy-dependent internalization of SiNPs was studied as a common negative control. We conducted these experiments at 4 °C, which sharply decreased the BCSC and MCF-7 uptake of SiNPs-OH, SiNPs-NH_2_, and SiNPs-COOH (concentration: 200 μg/mL), relative to the uptake and transport at 37 °C (Fig. 7a and b). The uptake inhibition of SiNPs-OH, SiNPs-NH_2_, and SiNPs-COOH was studied using flow cytometry. As shown in Fig. 7c, cyto D dramatically reduced uptake (>75%) in all treatment groups in MCF-7 and produced a much smaller effect in BCSCs (around 30%). Confocal results indicated that the actin of BCSCs demonstrated a thinner actin network with stress fibers, whereas in the present F-actin inhibitor (cyto D), the actin filaments and stress fibers were destroyed. Consequently, the uptake of SiNPs by cells was inhibited in the present of cyto D (Figure S7). In addition, negative internalization scores were obtained for CPZ in MCF-7 and BCSCs, which suggests low internalization after the treatment with this inhibitor. After the treatment with nystatin, nocodazole, genistein, and dynasore hydrate, the internalization value was similar to the positive control, which indicates no significant inhibition of SiNP internalization with these inhibitors (Fig. 7c–h). However, the BCSC uptake of SiNPs was sharply reduced because of the inhibition of the scavenger receptor *via* the Poly-I treatment. Similar results were obtained for PLAG nanoparticles and nanoliposomes (Figure S8).

**Figure 8:**
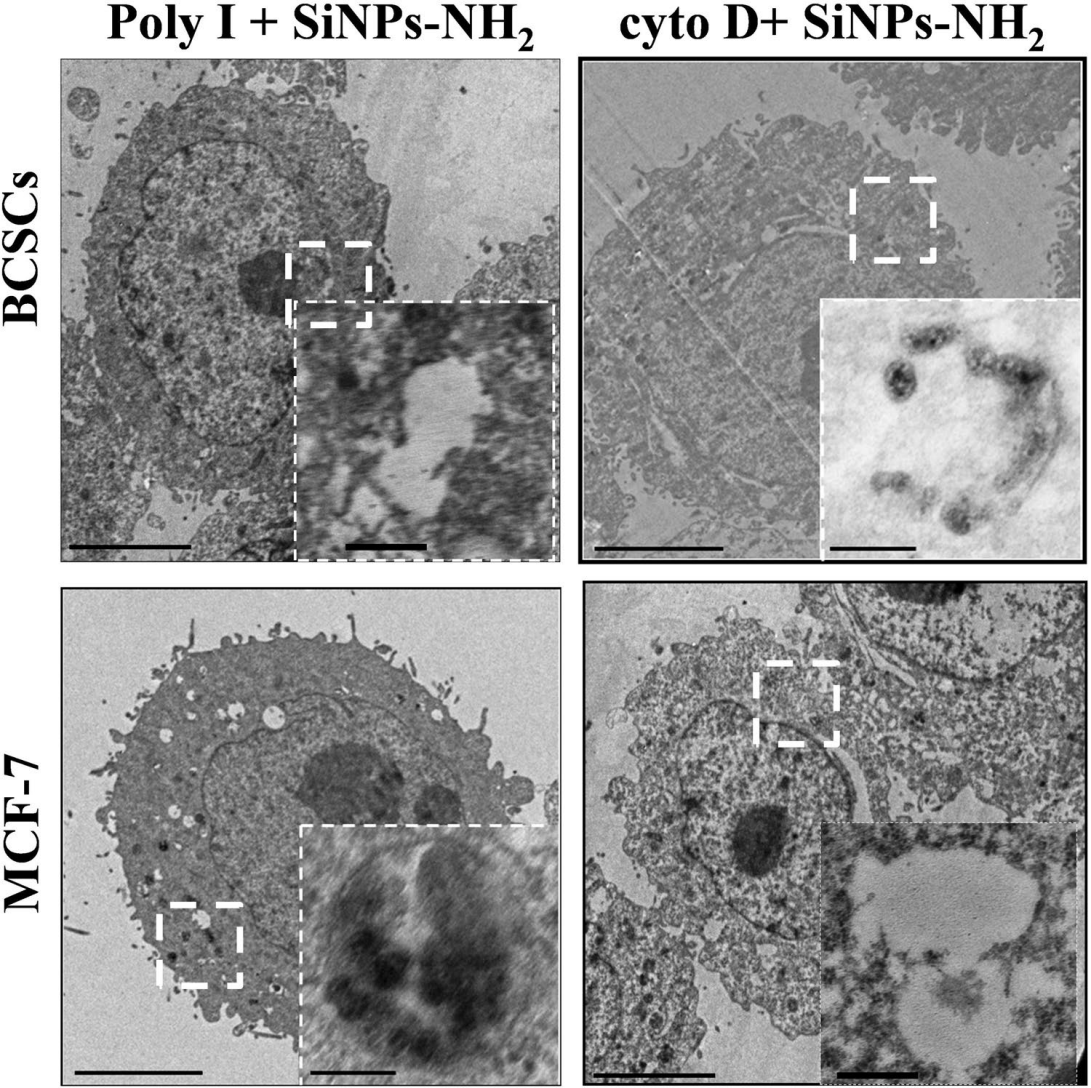
TEM images of the internalization inhibition of SiNPs-NH_2_ in MCF-7 and BCSCs after the Poly-I and cyto D treatments (scale bar: 5.0 μm). The magnified images of the area are indicated by the white box (scale bar, 500 nm).

**Figure 9:**
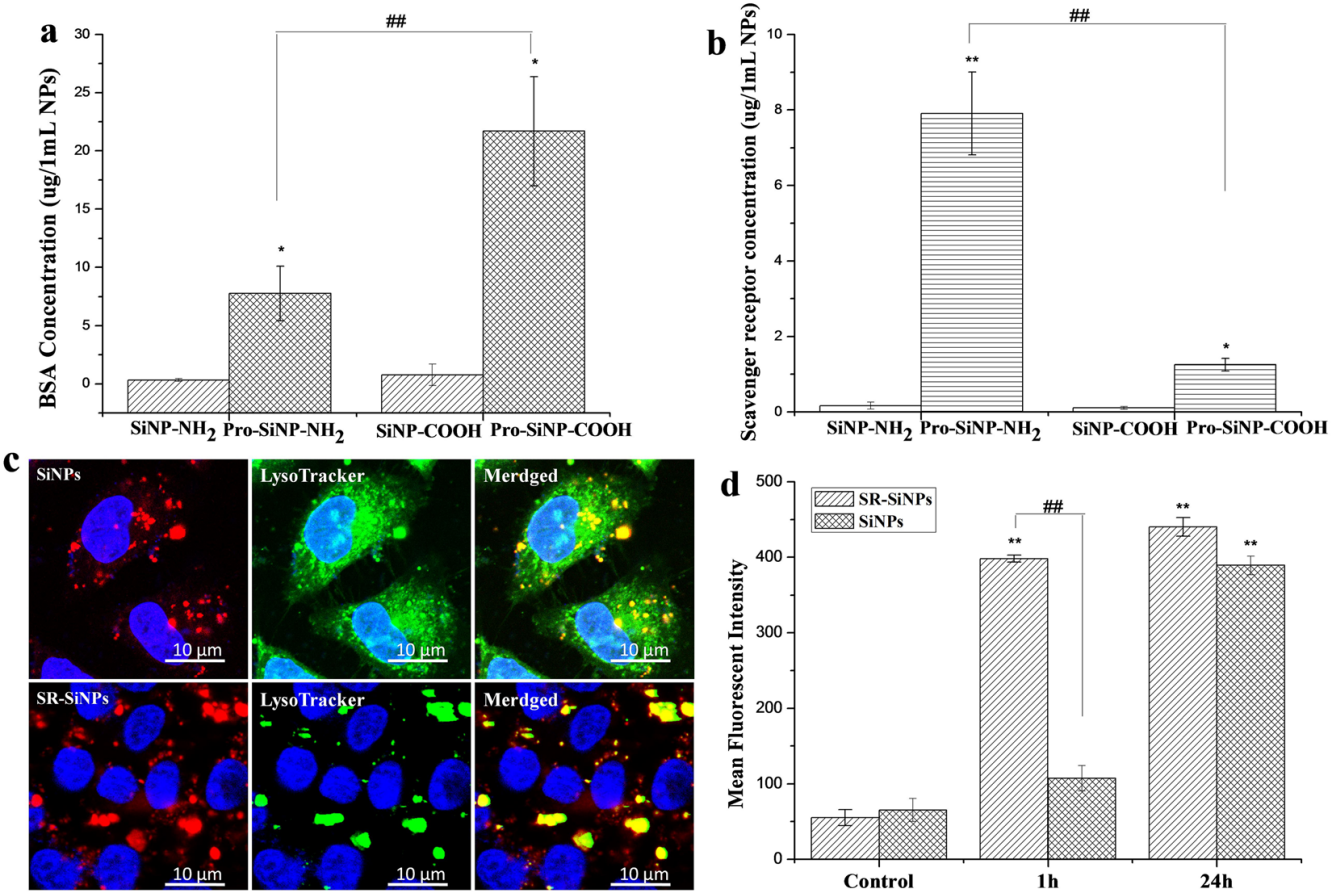
(**a**) BSA and (**b**) scavenger receptor content of protein-SiNP complexes. Protein-SiNP complexes formed from SiNPs-NH_2_ bind to scavenger receptors while complexes formed from SiNPs-COOH bind to native protein receptors. (**c**) Confocal images of the SiNPs-NH_2_ and SR-SiNPs-NH_2_ co-localized with lysosome in BCSCs after 1 h incubation. (**d**) MFI of at least 10,000 BCSCs, which was analyzed by FCM without or with SiNPs-NH_2_ and SR-SiNPs-NH_2_ treatment for 1 and 24 h. *p < 0.05; **p < 0.01 BCSCs versus MCF-7 in each group of SiNPs. ^##^p < 0.01 SiNPs-OH versus SiNPs-NH_2_ versus SiNPs-COOH in each cells type.

As shown in Fig. 8, the internalization of SiNPs-NH_2_ in BCSCs was significantly inhibited after the Poly-I treatment. For the cyto D treatment, notably few SiNPs-NH_2_ were observed in BCSCs, although the uptake of SiNPs-NH_2_ in BCSCs was sharply reduced. Conversely, the uptake of SiNPs-NH_2_ in MCF-7 was arrested by the treatment with cyto D but not Poly-I. This finding is consistent with the flow cytometry result in Fig. 7. To understand the role of scavenger receptor in the cellular uptake of SiNPs, we analyzed the expression of the scavenger receptor in BCSCs. The results indicated that the level of scavenger receptor was up-regulated after the SiNP treatment compared with MCF-7 (Figure S11). In addition, SiNPs-NH_2_ adsorb the proteins on to the surface when incubated in medium, forming an protein-SiNP s-NH_2_ complex (Figure S10), which are redirected to scavenger receptors (Fig. 9a and b). Thus, the SiNPs-NH_2_ mimic the complex structure that Poly-I forms to bind to scavenger receptors, which is activated for endocytosis. To confirm this finding, we conjugated the anti-scavenger receptor class B number 1(SCARB1) to the surface of SiNPs to study the efficiency of uptake in BCSCs. As shown in Fig. 9c and d, a significant uptake was observed after antiSR-SiNPs (100 μg/mL) at 1 h incubation compared to SiNPs, indicating a specific binding of SR-SiNPs to scavenger receptor, which promoted the uptake efficiency of NPs in a short time. This finding showed that scavenger receptor plays an important role in uptake of SiNPs in BCSCs, suggesting a rational design approach to target NPs to cancer stem cells.

Nanoparticles can localize the tumor site through the leaky blood vessel, which called the enhanced permeation and retention (EPR) effect. Two major methods are reported when nanocarriers target to tumour cells by EPR effect: passive targeting and active targeting. NPs whose surface is tailored (such as positive and negative charges) for prolonged blood circulation times, the concept is referred to as passive targeting. Targeting ligands, which bind to specific receptors on the tumour cells and endothelium, can be attached on the nanocarrier surface, this is called active targeting. Both passive and active nano-based drug delivery enhance the delivery efficiency of non-soluble or easily degradable therapeutic agents, and increase the therapeutic efficacy with minimal side effects through the different uptake mechanism (Figure S9).

## Discussion

Currently, increasingly many *in vitro* experiments to understand the endocytic mechanisms of NPs are typically conducted in either suspension cells or adherent cancer cells in nanomedicine-related fields for cancer treatment^21–23^. However, mechanisms by which NPs internalize the CSCs have been poorly defined. CSCs can more strongly initiate tumors and resist drugs than other cancer cells, which results in more inefficiency in CSC-targeted drug delivery. Therefore, we hypothesized that different internalization and transport processes were responsible for the cellular uptake in CSCs and cancer cell line. To test this hypothesis, a systematic study was present to understand the endocytic mechanisms of fluorescent-dye-loaded SiNPs-OH, SiNPs-NH_2_, and SiNPs-COOH in BCSCs and MCF-7, which provides an insight into the NP interaction with BCSCs, which may be different from other cancer cell lines. Figure 4 shows that the chemical groups play a prominent role in the uptake of NPs, where positively charged SiNPs-NH_2_ are taken up more readily than their negatively charged counterparts (SiNPs-OH and SiNPs-COOH) of identical diameters (Fig. 1). This result is perhaps not surprising considering that CSCs display a net negative surface charge because there are negatively charged extracellular plasma membrane protein moieties^24^. Therefore, the positively charged SiNPs-NH_2_ are expected to electrostatically interact with the plasma membrane, and this interaction may promote the SiNPs-NH_2_ association with the cells and induce the cell uptake *via* cellular endocytic mechanisms. Similarly, with the internalization of poly(ethylene glycol)-D, L-polylactide (PEG-PLA) NPs with a positive charge were twice as high as the internalization of PEG-PLA (negatively charged) NPs in polarized epithelial Madin-Darby Canine Kidney (MDCK) cells^25^. Since the NP uptake into cells can experience two major processes (phagocytosis and pinocytosis)^26^, we performed several studies using metabolic inhibitors. Phagocytosis mainly occurs in a small population of mammiferous cell types such as macrophages and monocytes. Pinocytosis is a common process has two subcategories: macropinocytosis and micropinocytosis. Macropinocytosis plays a major role in the non-specific uptake of soluble macromolecules. However, micropinocytosis happens for smaller particles in all cell types^27^. First, we showed that the internalization of SiNPs by CSCs was sensitive to temperature (Fig. 7a and b), which confirms the involvement of energy-dependent active processes. Then, we studied the cellular uptake mechanisms of SiNPs-OH, SiNPs-NH_2_, and SiNPs-COOH in MCF-7 and BCSCs by using seven endocytic inhibitors. Initially, we concentrated on three major pincytic uptake pathways: macropincytosis, clathrin-dependent, and caveolea/lipid raft-dependent micropinocytosis *via* treatment with nocodazole, CPZ and nystatin. There is no any uptake suppression was observed in MCF-7 and BCSCs for SiNP-OH, SiNP-NH_2_, and SiNP-COOH (Fig. 7d–f). The cytoskeleton plays a signigicant role in various cellular activities, including the formation of endocytosis vesicles in the endocytic progress. To investigate the function of the cytoskeletal reorganization on the SiNP uptaken in cells, we chose two inhibitors: cytochalasin D (cyto D) and nocodazole, which disturb the F-actin organization and microtubule formation, respectively^28^. As shown in Fig. 7c, for MCF-7, cyto D inhibited the endocytosis of SiNPs-OH, SiNPs-NH_2_, and SiNPs-COOH by approximately 80%, 85%, and 80% relative to the control, respectively. This result suggests that actin filaments are strongly involved in the endocytic precess of SiNPs and particularly SiNPs-NH_2_ in the MCF-7 cell line. However, only approximately 20% reduction for SiNPs uptake in BCSCs was observed, indicating a less F-actin-dependent uptake of SiNP for BCSCs. The result can be explained by a different inhibition efficiency induced by cyto D in different cell lines. It has reported that microtubules play a key role in the process of endocytosis, and nacodazole disrupts the microtubule. As shown in Fig. 7f, only 5% inhibition rate of SiNPs-COOH was obtained relative to the control group in MCF-7, and no inhibition of uptake of other SiNPs was observed in BCSC. Thus, microtubules did not involve in the uptake of SiNPs in MCF-7 and BCSCs. It has reported that the clathrin-dependent pathway can regulate the uptake of SiNPs^29^. In this study, chlorpromazine (CPZ), which is an inhibitor of clathrin-mediated encytosis, was used to investigate the internalization of SiNPs. The data showed that the SiNP uptake by MCF-7 and BCSCs slightly decreased after the clathrin inhibition (Fig. 7d). However, the non-uptake inhibition was obtained for SiNPs in the cells treated with nystatin, which indicated that the uptake of SiNPs is irrelevant to the lipid raft-dependent uptake mechanisms (Fig. 7e). Lately, the dynamin-dependent endocytic mechanism has been widely studied as a possible clathrin-independent endocytic pathway into cells^30^. However, our results did not have any significant changes of cellular uptake after treatment with dynasore, which disrupts the dynamin-dependent pathways. These results indicate that macropinocytosis, clathrin-dependent endocytosis, and lipid raft-dependent endocytosis are not the major entry mechanisms of SiNPs in MCF-7 and BCSCs. To evaluate whether a more specific endocytic mechanism is involved for the SiNP uptake in MCF-7 and BCSCs, we discussed another two uptake pathways in MCF-7 and BCSCs, including the scavenger receptors and membrane-bound G-protein coupled receptor (GPCR)-mediated uptake. Scavenger receptors have been reported to be involved in AuNP uptake through binding to various ligands, such as low-density lipoproteins and polysaccharides^31^. Poly-I, which is a widely accepted inhibitor of scavenger receptors, supressed the SiNP enter into BCSCs (Figs 7i and 8), which confirms the function of scavenger receptors in the endocytic process of SiNP in different cell lines. In this work, the uptake inhibition was significantly inhibited for SiNPs in breast cancer stem cells treated with Poly-I, but no similar results was observed in MCF-7 cells. All results with different endocytic pathways inhibitors in MCF-7 and BCSCs are summarized in Table 1.

**Table 1:** Summary of the uptake inhibition of SiNPs in the presence of endocytic inhibitors.

Inhibitor	Function	SiNP-OH		SiNP-NH_2_		SiNP-COOH	
		MCF-7	BCSC	MCF-7	BCSC	MCF-7	BCSC
cyto D	Inhibits F-actin polymerization	++	+	++	+	++	+
Nocodazole	Disruption of microtubules	−	−	−	−	−	−
CPZ	Inhibits clathrin	+	+	+	+	+	+
Dynasore	Inhibits dynamin-GTPase	−	−	−	−	−	−
Nystatin	Lipid-raft inhibitor	−	−	−	−	−	−
Poly I	Scavenger receptor inhibitor	−	++	−	++	−	++
Genistein	Inhibitor of several tyrosine kinases	−	−	−	−	−	−

+p < 0.05, ++p < 0.01 through unpaired t-test between control and inhibitor-treated groups. − no significant inhibition.

**Figure 10:**
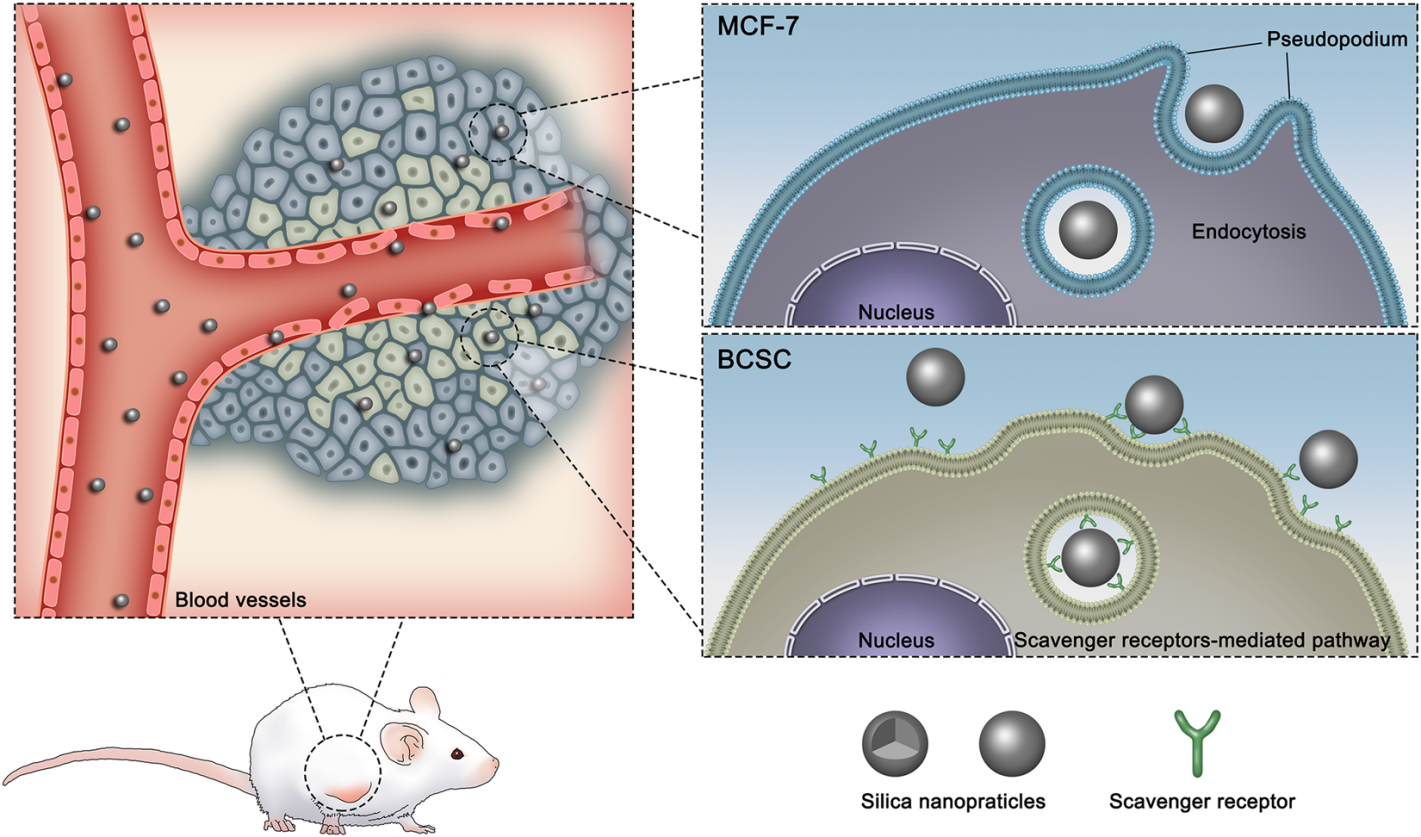
Schematic model to illustrate different endocytic mechanisms of SiNPs in breast cancer cells and BCSCs.

Overall, a large range of endocytic pathways, i.e., macropinocytosis, clathrin-mediated endocytosis, and caveplae/lipid raft-mediated endocytosis, are not involved in the uptake for SiNPs in MCF-7 and BCSCs. However, for the MCF-7 cell line, strongly mediated uptake was observed only *via* actin filament endocytosis. For BCSCs, both actin filament endocytosis and scavenger receptor-induced uptake were primary for all SiNPs. The actin is a major part of the cytoskeleton and a large number of evidence showed that F-actin plays a vital role in the process of internalization. Recent microscopy studies provide strong evidence that formation and organization of actin occurs at endocytic vesica formation sites, where create protrusions that encompass extracellular materials^18,32–35^. In this work, we evaluated how the scavenger receptor facilitates the SiNPs uptake into BCSCs through inhibited the expression of F-actin. The results indicated that the actin of BCSCs demonstrated a thinner actin network with stress fibers, whereas in the present F-actin inhibitor (cyto D), the actin filaments and stress fibers were destroyed. Consequently, the uptake of SiNPs by cells was inhibited in the present of cyto D. It has reported that several proteins (including SCAR/WASP) are directly participated in mediating actin organization and could thus control forces production during actin organization to promote specific steps in the endocytosis^36–38^. The expression of the scavenger receptor in BCSCs was up-regulated after the SiNP treatment compared with MCF-7 (Figure S11). In addition, both cationic and anionic SiNPs adsorb the proteins on to the surface when incubated in medium, forming an anionic protein-SiNP complex (Figure S10). We determined that protein-SiNP-COOH complexes bind to albumin receptors on the cell surface. Protein-SiNP-NH_2_ complexes are redirected to scavenger receptors (Fig. 9a and b). Thus, the SiNPs mimic the complex structure that Poly-I forms to bind to scavenger receptors, which is activated for endocytosis. Indeed, the reduction in density of gold nanoparticles reportedly decreases the Poly-I-dependent inhibition of NP uptake^19,31^. In addition, the chemical groups of SiNPs play a crucial role in terms of dictating their interaction with the cells. The positively charged SiNPs-NH_2_ more efficiently associate with the cells than the negatively charged SiNPs-OH and SiNPs-COOH and consequently induce the cell uptake *via* cellular endocytic mechanisms. A schematic model to illustrate different endocytic mechanisms of SiNPs in breast cancer cells and BCSCs is shown in Fig. 10.

## Conclusion

In conclusion, SiNPs can internalize in notably different cells through different endocytic pathways. This study highlights the complexity and interaction among the endocytic mechanisms by which different cell lines can uptake SiNPs and suggests that the SiNPs with the same functionalization may be uptaken *via* different endocytic mechanisms in MCF-7 and BCSCs. We suggest that for MCF-7 cells, the uptake of SiNPs was inhibited by treatment with Cyto D, indicating a F-actin-dependent pathway. In contrast, SiNPs are internalized by BCSCs *via* a different route that involves the scavenger-receptor-mediated endocytosis pathway. Importantly, positively charged SiNPs have higher cell uptake than negatively charged SiNPs in both MCF-7 and BCSCs, which is important in designing drug carriers that can cross the mucosal barriers and enable a noninvasive delivery of biological therapeutics. In addition, the presence of different endocytic mechanisms for functionalized SiNPs in tumor cells and CSCs provides a valuable platform to design nanocarriers of cancer-stem-cell-targeted therapeutics.

## Methods

### Materials

Tetraethyl orthosilicate (TEOS) and tris(2,2′-bipyridyl)dichlororuthenium(II) hexahydrate (Rubpy) was obtained from Acros Organics. Triton X-100 and 3-aminopropyl trimethoxysilane (APTES) were obtained from Acros. Carboxyethylsilanetriol sodium salt (CTES) (25 wt.% in water) was bought from Sigma, USA. Genistein, nystatin, chlorpromazine hydrochloride (CPZ), cyto D, nocodazole, dynasore and Poly-I were obtained from Sigma-Aldrich. The cell culture reagents were purchased from HyClone. The used reagents are of analytical grade. Anti-CD44-FITC, CD133-PE, and anti-Scavenger Receptor were obtained from Invitrogen.

### Synthesis of Dye-loaded Silica Nanoparticles

SiNPs were synthesized by a water-in-oil (W/O) microemulsion method. Briefly, cyclohexane (7.5 mL), Triton X-100 (1.77 mL) and hexanol (1.8 mL) were mixed into the round-bottom flask with string for 10 min to compose the microemulsion system; then, H_2_O (340 μL), Ru(bpy)_3_Cl_2_ dye (80 μL of 0.1 M), and TEOS (100 μL) were added. Then the mixture was added 100 μL of aqueous ammonia (28–30 wt%) and stirred for 24 h at room temperature to complete the hydrolysis reaction. Finally, acetone was introduced to break the microemulsion; After centrifugation, the SiNPs were obtained and sequentially washed three times with acetone and deionized water.

### Synthesis of Functionalized Silica Nanoparticles

The amino-group-functionalized SiNPs (SiNPs-NH_2_) were synthesized according to the reported method^20^. Briefly, 50 mg SiNPs was dispersed to 20 mL toluene by magnetic stirring; then, 50 μL APTES was added during the nitrogen protection; the reaction was continued for 24 h stirring. Subsequently, the SiNPs-NH_2_ were washed ethanol and deionized water for three times. The carboxyl group functionalized SiNPs (SiNPs-COOH) was synthesized according to the reported method^20^. In brief, 50 μL APTES and 0.02 mg succinic anhydride evenly dispersed in 20 mL dimethyl formamide for 3 h of stirring at 37 °C. Then, 20 mL SiNPs-NH_2_ by dimethyl formamide ultrasonic dispersion and 2 mL deionized water were added into the system and continuously stirred for 5 h. Finally, the SiNPs-COOH were washed with ethanol and deionized water for three times.

### Characterization of Functionalized SiNPs

The diameter and morphology of the SiNPs were studied using a scanning electron microscope (SEM, Hitachi), a transmission electron microscope (TEM, Philips CM12). The size of SiNPs was studied by dynamic light scattering (DLS). The DLS values and zeta potential were tested through a DLS Particle Size Analyzer (Malvern ZetasizerNano ZS, UK). The fluorescence spectra were obtained at room temperature using a fluorescence spectrophotometer (Hitachi F-4600, Japan). The surface groups of carboxyl and amino were characterized by infrared spectroscopy (IR, ThermoFisher).

### Isolation of MCF-7-derived Breast Cancer Stem Cells

Breast cancer cell line (MCF-7) was purchased from ATCC and cultured in Dulbecco’s Modified Eagle’s Medium (DMEM, with additional L-Glutamine) in present with 10% fetal bovine serum (FBS, Gibco) and 1% penicillin/streptomycin (Gibio) at the condition of 37 °C and 5% CO_2_. The culture medium was changed every three days.

BCSCs were isolated from the MCF-7 cell line and grown in a DMEM/F12 medium (Gibco) in presence of 20 ng/mL EGF, 10 ng/mL bFGF, 2% B27, 1% N2 (Invitrogen, USA), and 1% penicillin/streptomycin. Then, the 5,000 cells were seeded and incubated in ultra-low-attachment 6-well plates (Corning, USA) to form the tumor sphere.

### Tumor Sphere Formation and Passage Assay

After gentle centrifugation, the tumor spheres were concentrated, and then digested with trypsin-EDTA and mechanically separated with a pipette. The digested single cells at a density of 5000 cells/well were washed with PBS and centrifuged to remove the trypsin-EDTA and re-seeded in a medium supplemented with growth factors to re-form tumor spheres. The re-formed spheres must be passaged every 10–12 days when the diameter reached to 200 μm.

### Identification of BCSCs

For the surface marker analysis by confocal microscopy, the tumor spheres were digested from the culture plates using trypsin-EDTA (Invitrogen). Single MCF-7 and BCSCs were washed with PBS and stained with CD44-FITC and CD133-PE antibodies according to the manufacture. The results were monitored using a laser scanning confocal microscope (LSCM, ZEISS).

### *In vivo* Tumorigenesis of BCSCs

BCSCs and MCF-7 cells were suspended in 100 μL sterile PBS at the density of 5000 cells and subcutaneously injected in the back of the nude mice. The mice were measured every five days for tumor growth for up to 30 days. All animal experiments were carried followed with the laboratory animal care and the guide for the care and use of laboratory animals. All experimental protocols were authorized by the administration office committee of laboratory animal of Hebei University.

### *In vitro* Cytotoxicity of SiNPs

The viability of cells cultured with SiNPs was observed by 4,5-(dimethyl-2-thiazolyl)-2,5-diphenyl-2-H-tetrazolium bromide (MTT) assay^23^. SiNP solutions (20, 50, 100, and 200 μg/mL) incubated with cells for 24, 48, and 72 h, and then the medium was added 10 μL of MTT solution. The optical density (OD) of the medium at 570 nm was recorded with a microplate reader. The data are expressed as follows: (OD_sample_ − OD_blank_)/(OD_control_ − OD_blank_) × 100%.

### Treatments with Chemical Inhibitors

For the experiment of endocytic pathways suppression, 5 × 10^4^ cells were pre-treated with different endocytic inhibitors for 30 min at the following concentrations: genistein (10 μg/mL); Dynasore (20 μM); cyto D (5 μg/mL); polyinosinic acid potassium salt (Poly-I, 10 μg/mL); nocodazole (10 μg/mL); nystatin (10 μg/mL); CPZ (10 μg/mL). After these pre-treatments, SiNPs were introduced to the cell culture medium and incubated for 4 h. Then, the cells were washed three times with PBS and stored in darkness at 4 °C before the measurement.

### Flow Cytometry Analysis of SiNP Uptake

The cells (5 × 10^4^ cells/well) were cultured on 6-well plates and added SiNPs when they had reached 70–80% confluence. The medium was removed, and the uptake medium containing 200 μg/mL SiNPs was introduced into cells and continued to culture for 4 and 24 h. The cellular uptake and inhibition of SiNPs were determined by flow cytometry (BD CantoII, USA).

### SiNPs Uptake Using a Confocal Microscope

The cellular uptake and localization of fluorescent-dye-loaded SiNPs was imaged using a confocal microscope (ZEISS, LSM880, Germany). The cells (4 × 10^4^ cells/well) were treated with 200 µg/mL SiNPs, and then were fixed in 4% PFA for 15 min at room temperature and washed with PBS for 3 times. The nucleus was stained with Hoest33342. The cells were mounted with a cover slip for the confocal microscope.

### Analysis of the Cellular Uptake and Location of SiNPs by TEM

4 × 10^4^ cells were incubated with 200 μg/mL SiNPs for 24 h. Then, the cells were first fixed in 2% buffered in 0.1 M sodium cacodylatefor 2 h. The samples were post-fixed with 2% OsO_4_ in the dark for 2 h. The samples were dehydrated through alcohol with gradient concentrations (10, 50, 70, 90, 95, and 100%) and further acetone dehydration (90, 96, and 100%). Finally, the samples were treated with propylene oxide: resin (1:1) overnight. Then, the ultra-thin sections (80 nm) was obtained and viewed under a TEM at 80 kV (Philips Tecnai 12 BioTWIN).

### Western Blot

BCSCs and MCF-7 cells (2 × 10^4^ cells/well) were treated with SiNPs overnight in a normal cell culture medium. Then, the cells were rinsed 3 times with cold PBS and lysed in protein lysate. The collected protein from each sample was separated by 10% SDS-PAGE gel and transferred onto nitrocellulose membranes, which were blocked in a buffer contained 5% BSA in a TBST solution for 2 h and hybridized with anti-scavenger receptor overnight at 4 °C, and then incubated with peroxidase-conjugated secondary antibody and interacted with a chemoluminescence test kit (Thermo Fisher Scientific). *β*-Actin was used as the loading control.

### Elisa assay

The SiNPs were incubated with cells in normal culture medium with 10% FBS for 24 h to evaluate the ability of protein absorption. Then, the zeta potential of protein-SiNPs was measured by DLS Particle Size Analyzer. The concentration of BSA and scavenger receptor was tested by using Bio-rad protein assay (Bio-rad) and elisa assay (Beyotime Biotechnology, China) according to the protocol.

### Statistical Analysis

Each experiment was performed three times in quadruplicate. The statistical analysis was analyzed using Student’s t-test and analysis of variance (one-way ANOVA) followed by Bonferroni post hoc test for multiple comparisons, and the results were presented as the mean ± SD. Statistical significance was accepted at a level of p < 0.05.

### Data Availability

All data generated or analysed during this study are included in this published article (and its Supplementary Information files).

## Electronic supplementary material

supporting information
