# Supplementary material for: A Distinct Endocytic Mechanism of Functionalized-Silica Nanoparticles in Breast Cancer Stem Cells
Source: Sci Rep. 2017 Nov 24;7:16236. doi: 10.1038/s41598-017-16591-z (PMC5701218; doi:10.1038/s41598-017-16591-z)
Supplement: Supplementary file 1 — supporting information [file 41598_2017_16591_MOESM1_ESM.doc]

**Supporting Information**

**A Distinct Endocytic Mechanism of Functionalized-Silica Nanoparticles in Breast Cancer Stem Cells**

Jiadong Sun ^1,2,3,+^, Yajing Liu ^1,2, +^, Min Ge ^1,2^ , Guoqiang Zhou ^1,2^, Wentong Sun ^1,2^, Dandan Liu ^1,2^, *, Xing-Jie Liang ^3^,*, Jinchao Zhang ^1,2^,*

1 Key Laboratory of Medicinal Chemistry and Molecular Diagnosis of the Ministry of Education, Hebei University, Baoding 071002, People’s Republic of China

2 College of Chemistry and Environmental Science, Chemical Biology Key Laboratory of Hebei Province, Hebei University, Baoding 071002, People’s Republic of China

3 CAS Key Laboratory for Biological Effects of Nanomaterials and Nanosafety, National Center for Nanoscience and Technology, Beijing 100190, People’s Republic of China

* Corresponding author: Dandan Liu, Jinchao Zhang, and Xing-Jie Liang

*Address correspondence to [leo-liudan@163.com](mailto:leo-liudan@163.com); [liangxj@nanoctr.cn](mailto:liangxj@nanoctr.cn) and [jczhang6970@163.com](mailto:jczhang6970@163.com)

**^+^** These authors contributed equally to this work.

**Results**

**IR characterization of dye-loaded functionalized-SiNPs**

The amino- and carboxyl-functionalized SiNPs were characterized by IR. At 1479 cm^-1^ respectively showed amino symmetric bending vibration absorption peak and vibration absorption peak, it is fully demonstrated after grafting amino groups can be successfully grafted to the surface of the SiNPs (Figure S1b). In addition, amino N-H alive in 2923 cm^-1^ has obvious absorption band, the above analysis shows that the chemical bonds on the surface of the silica successfully modified amination. As shown in Figure S1c, at 462 cm ^-1^ and 1081 cm ^-1^ has obvious absorption peak is Si-O-Si transverse and longitudinal stretching vibration peak. In addition, free carboxyl between 1700-1725 cm ^-1^ with carbonyl stretching vibration peak into carboxyl functionalized SiNPs and secondary amide in 1555-1642 cm ^-1^ vibration peak, our infrared at 1555 cm ^-1^ has obvious vibration peak, shows that the experiment success of nano silicon dioxide modified material structure and properties of silica and there is no change.

Figure S1 Determination of (a) hydroxyl, (b) amino groups, and (c) carboxyl groups of functionalized-SiNPs by IR.

***In vitro* cytotoxicity of SiNPs**

Cytotoxicity was tested on MCF-7 and BCSCs that were respectively treated with varying doses of SiNPs-OH, SiNPs-NH_2_, and SiNPs-COOH (25, 50, 100, and 200 μg/mL) for 24 h. Cell viability determined by the MTT assay showed that a dosage level lower than 200 μg/mL did not show significant cytotoxicity in MCF-7 and BCSCs (Figure S2). Therefore, the dosage range of 25-200 μg/mL of SiNPs-OH, SiNPs-NH_2_, and SiNPs-COOH and 24 h exposure were selected for further studies.

Figure S2 Cytoxicity of SiNPs-OH, SiNPs-NH_2_, and SiNPs-COOH in (a) MCF-7 and (b) BCSCs. Cells were treated with various concentrations of SiNPs for 24 h and the viability was determined by MTT. Control cells cultured in NPs-free medium were run in parallel to the treated groups. Values were the mean ± SD from three independent experiments. Signiﬁcance indicated by: *p < 0.05 versus control cells.

**Amount internalization of SiNPs**

Figure S3 illustrates that cellular internalization of positively charged SiNPs-NH_2_ in MCF-7 (Figure S3a) and BCSCs (Figure S3b) is significantly higher compared to SiNPs with negatively charged surface at all applied doses. Cell internalization in general increased with dose. Interestingly, this increase appears to be steeper for negatively charged SiNPs-NH_2_ in comparison to positively charged systems indicating a higher internalization dose dependence for negatively charged nanoparticles compared to positively charged nanoparticulates, even though the latter displayed a comparatively higher cell uptake at all concentrations.

Figure S3 Internalization of SiNP-NH_2_, SiNP-OH, and SiNP-COOH by (a) MCF-7 and (b) BCSCs. (c) The uptake ratio of SiNPs in BCSCs relative to MCF-7. Data represents the mean ± standard deviation (n = 5). *p < 0.05, **p <0.01 vs control.

**SiNPs** **localization in lysosome**

Figure S4 Magnification images of SiNPs-OH, SiNPs-NH_2_, and SiNPs-COOH co-localized with lysosome in (a) MCF-7 and (b) BCSCs.

Figure S5 3D images show the SiNPs-OH, SiNPs-NH_2_, and SiNPs-COOH co-localized with lysosome in (a) MCF-7 and (b) BCSCs.

Figure S6 Colocalized SiNPs with lysotracker. The results indicated that the overlap rate of SiNPs with lysotracker reached to 83% after (a) 4 h incubation. The overlap rate of SiNPs with lysotracker reduced to around 70% and 49% after (b) 8 h and (c) 12 h incubation, respectively. This finding showed that the SiNPs firstly entered the cells and localized in lysosomes, and then the SiNPs escaped into the cytoplasm.

**Inhibition of uptake in BCSCs in present of cyto D**

Figure S7 Uptake of SiNPs in BCSCs in the present cyto D. The actin of BCSCs demonstrated a thinner actin network with stress fibers, whereas in the present F-actin inhibitor (cyto D), the actin filaments and stress fibers were destroyed. Consequently, the uptake of SiNPs by cells was inhibited in the present of cyto D.

**Uptake of liposome and PLGA NPs in BCSCs**

Figure S8 Representative TEM images of PLGA NPs (a) and nanoliposome (b). The percentage of cellular uptake of PLGA NPs (c) and liposomes (d) in MCF-7 and BCSCs in the presence of different endocytic inhibitors. *p < 0.05, **p <0.01 vs control.

**Uptake of CD133-conjugated SiNPs in BCSCs**

Figure S9 showed that the uptake rate was obviously decreased when BCSCs in presence of chlorpromazine (CPZ) which promotes clathrin assembly and suppresses the receptor recycling to the cell membrane during CME. These findings demonstrated that CD133-SiNPs bind to specific receptors expressed on the BCSCs and endothelium and internalized by BCSCs *via* a clathrin-mediated endocytosis pathway.

Figure S9 The percentage of cellular uptake of CD133-conjugated SiNPs in MCF-7 and BCSCs in the presence of different endocytic inhibitors. Data represents the mean ± standard deviation (n = 5). *p < 0.05, **p <0.01 vs control.

**Zeta potential of protein-SiNPs**

Figure S10 Zeta potential of protein-SiNPs. Serum proteins adsorb onto the surface of both cationic and anionic SiNPs, forming a net anionic protein-SiNP complex.

**High Expression of Scavenger Receptor in BCSCs**

MCF-7 and BCSCs were incubated with 200 μg/mL SiNPs for 24 h before lysis of the cells. As shown in Figure S11, the scavenger receptor expression in BCSCs was up-regulated after SiNPs treatment compared with MCF-7.

Figure S11 High expression of scavenger receptor in BCSCs after SiNPs treatment for 24 h was monitored by Western Blotting (a) and q-PCR (b). *p < 0.05 BCSCs vs. MCF-7. The bands of scav R and β-actin were cropped from different gels, which were separated by white space. The images were obtained through BIO-RAD Gel Doc XR+ system (Bio-Rad, USA) without touch-up and overexposure. The original images were present in Supplementary Figure S12.

Figure S12 Original images of Western Blot. (a) Scav R (upper), β-actin (lower), (b) β-actin. Due to the overexposure of β-actin in (a), we re-imaged the bands and present in (b).
